# 5-[3-(2,5-Dimethoxy­phen­yl)prop-2-enyl­idene]-1,3-diethyl-2-thioxohexa­hydro­pyrimidine-4,6-dione

**DOI:** 10.1107/S1600536809026099

**Published:** 2009-07-11

**Authors:** Abdullah Mohamed Asiri, Salman A. Khan, Seik Weng Ng

**Affiliations:** a Chemistry Department, Faculty of Science, King Abdul Aziz University, Jeddah, Saudi Arabia; b Department of Chemistry, University of Malaya, 50603 Kuala Lumpur, Malaysia

## Abstract

1,3-Diethyl-2-thio­barbituric acid reacts with 2,5-dimethoxy­benzaldehyde to form the title Knoevenagel product, C_19_H_22_N_2_O_4_S. In the compound, the two six-membered rings at either end of the three-membered –CHCHCH– chain are nearly coplanar with this fragment (r.m.s. deviation of the two six-membered rings and the three chain atoms = 0.08 Å).

## Related literature

For the reaction of 1,3-diethyl-2-thio­barbituric acid with aromatic aldehydes to form the Knoevenagel and Michael products, see: Adamson *et al.* (1999 ▶).
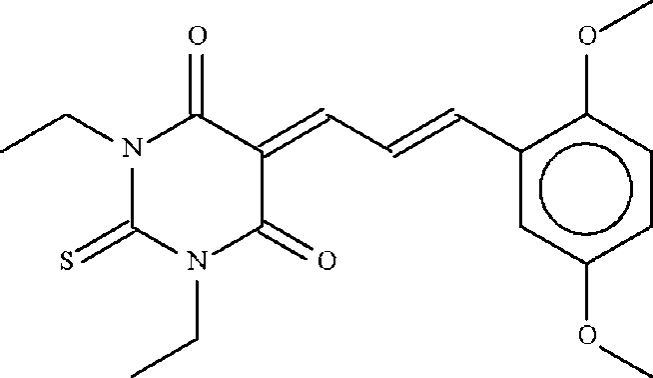


## Experimental

### 

#### Crystal data

C_19_H_22_N_2_O_4_S
*M* *_r_* = 374.45
Monoclinic, 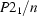

*a* = 10.0519 (2) Å
*b* = 15.5942 (3) Å
*c* = 11.5920 (2) Å
β = 90.813 (1)°
*V* = 1816.88 (6) Å^3^
*Z* = 4
Mo *K*α radiation
μ = 0.21 mm^−1^
*T* = 140 K
0.35 × 0.25 × 0.15 mm

#### Data collection

Bruker SMART APEX diffractometer
Absorption correction: multi-scan (*SADABS*; Sheldrick, 1996 ▶) *T*_min_ = 0.932, *T*_max_ = 0.970
12384 measured reflections
4124 independent reflections
3351 reflections with *I* > 2σ(*I*)
*R*_int_ = 0.021

#### Refinement

*R*[*F* ^2^ > 2σ(*F* ^2^)] = 0.039
*wR*(*F* ^2^) = 0.119
*S* = 1.02
4124 reflections
239 parameters
H-atom parameters constrained
Δρ_max_ = 0.38 e Å^−3^
Δρ_min_ = −0.31 e Å^−3^

### 

Data collection: *APEX2* (Bruker, 2008 ▶); cell refinement: *SAINT* (Bruker, 2008 ▶); data reduction: *SAINT*; program(s) used to solve structure: *SHELXS97* (Sheldrick, 2008 ▶); program(s) used to refine structure: *SHELXL97* (Sheldrick, 2008 ▶); molecular graphics: *X-SEED* (Barbour, 2001 ▶); software used to prepare material for publication: *publCIF* (Westrip, 2009 ▶).

## Supplementary Material

Crystal structure: contains datablocks global, I. DOI: 10.1107/S1600536809026099/xu2548sup1.cif

Structure factors: contains datablocks I. DOI: 10.1107/S1600536809026099/xu2548Isup2.hkl

Additional supplementary materials:  crystallographic information; 3D view; checkCIF report

## Figures and Tables

**Fig. 1.**
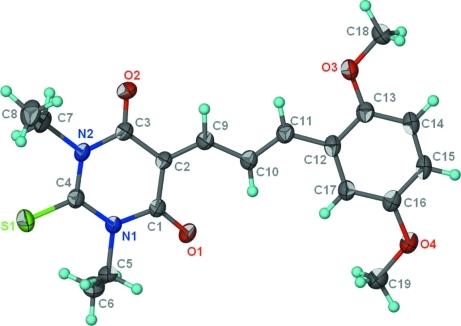
Thermal ellipsoid plot (Barbour, 2001) of C19H22N2O4S at the 70% probability level; hydrogen atoms are drawn as spheres of arbitrary radius.

## supplementary crystallographic information

### Experimental

1,3-Diethyl-2-thiobarbituric acid (1 g, 0.005 mol) and 2,5-dimethoxybenzaldehyde (0.83 g, 0.005 mol) were heated in ethanol (15 ml) for 3 h; several drops of pyridine were added. The progress of reaction was monitored by TLC. The solid that seperated from the cool mixture was collected and recrystallized from a methanol-chloroform mixture in 50% yield; m.p. 454 K.

### Refinement

Carbon-bound H-atoms were placed in calculated positions (C—H 0.95 to 0.99 Å) and were included in the refinement in the riding model approximation, with *U_iso_*(H) fixed at 1.2–1.5*U_eq_*(C).

### Crystal data

**Table:**

C_19_H_22_N_2_O_4_S	*F*(000) = 792
*M**_r_* = 374.45	*D*_x_ = 1.369 Mg m^−^^3^
Monoclinic, *P*2_1_/*n*	Mo *K*α radiation, λ = 0.71073 Å
Hall symbol: -P 2yn	Cell parameters from 4985 reflections
*a* = 10.0519 (2) Å	θ = 2.2–28.3°
*b* = 15.5942 (3) Å	µ = 0.21 mm^−^^1^
*c* = 11.5920 (2) Å	*T* = 140 K
β = 90.813 (1)°	Irregular, gold–green
*V* = 1816.88 (6) Å^3^	0.35 × 0.25 × 0.15 mm
*Z* = 4	

### Data collection

**Table:**

Bruker SMART APEX diffractometer	4124 independent reflections
Radiation source: fine-focus sealed tube	3351 reflections with *I* > 2σ(*I*)
graphite	*R*_int_ = 0.021
ω scans	θ_max_ = 27.5°, θ_min_ = 2.2°
Absorption correction: multi-scan (*SADABS*; Sheldrick, 1996)	*h* = −12→13
*T*_min_ = 0.932, *T*_max_ = 0.970	*k* = −20→19
12384 measured reflections	*l* = −15→15

### Refinement

**Table:**

Refinement on *F*^2^	Primary atom site location: structure-invariant direct methods
Least-squares matrix: full	Secondary atom site location: difference Fourier map
*R*[*F*^2^ > 2σ(*F*^2^)] = 0.039	Hydrogen site location: inferred from neighbouring sites
*wR*(*F*^2^) = 0.119	H-atom parameters constrained
*S* = 1.02	*w* = 1/[σ^2^(*F*_o_^2^) + (0.0635*P*)^2^ + 0.8319*P*] where *P* = (*F*_o_^2^ + 2*F*_c_^2^)/3
4124 reflections	(Δ/σ)_max_ = 0.001
239 parameters	Δρ_max_ = 0.38 e Å^−^^3^
0 restraints	Δρ_min_ = −0.31 e Å^−^^3^

### Fractional atomic coordinates and isotropic or equivalent isotropic displacement parameters (Å^2^)

**Table:**

	*x*	*y*	*z*	*U*_iso_*/*U*_eq_	
S1	0.45514 (5)	0.17006 (3)	0.48043 (5)	0.04028 (15)	
O1	0.65557 (12)	0.44794 (7)	0.58487 (12)	0.0369 (3)	
O2	0.88016 (13)	0.19441 (7)	0.68934 (12)	0.0368 (3)	
O3	1.30863 (11)	0.51465 (7)	0.76778 (11)	0.0323 (3)	
O4	1.02427 (13)	0.79694 (8)	0.61792 (13)	0.0412 (3)	
N1	0.57177 (12)	0.31723 (8)	0.53660 (11)	0.0229 (3)	
N2	0.67570 (13)	0.19089 (8)	0.60462 (11)	0.0238 (3)	
C1	0.67099 (15)	0.37060 (9)	0.58438 (13)	0.0242 (3)	
C2	0.78972 (15)	0.32772 (9)	0.63022 (13)	0.0229 (3)	
C3	0.78870 (16)	0.23434 (9)	0.64445 (13)	0.0250 (3)	
C4	0.57229 (15)	0.22878 (10)	0.54306 (14)	0.0248 (3)	
C5	0.45950 (15)	0.36105 (10)	0.47751 (14)	0.0265 (3)	
H5A	0.4319	0.3274	0.4088	0.032*	
H5B	0.4890	0.4182	0.4508	0.032*	
C6	0.34165 (17)	0.37168 (11)	0.55619 (16)	0.0341 (4)	
H6A	0.2705	0.4026	0.5152	0.051*	
H6B	0.3690	0.4042	0.6249	0.051*	
H6C	0.3092	0.3151	0.5794	0.051*	
C7	0.67507 (17)	0.09673 (10)	0.62395 (15)	0.0303 (4)	
H7A	0.5820	0.0766	0.6301	0.036*	
H7B	0.7216	0.0837	0.6977	0.036*	
C8	0.7422 (2)	0.04915 (11)	0.52713 (19)	0.0415 (4)	
H8A	0.7466	−0.0121	0.5458	0.062*	
H8B	0.8325	0.0715	0.5175	0.062*	
H8C	0.6911	0.0572	0.4553	0.062*	
C9	0.90368 (16)	0.36954 (10)	0.66047 (13)	0.0250 (3)	
H9	0.9718	0.3342	0.6923	0.030*	
C10	0.93689 (16)	0.45823 (10)	0.65184 (13)	0.0254 (3)	
H10	0.8741	0.4981	0.6216	0.031*	
C11	1.05873 (16)	0.48485 (10)	0.68724 (14)	0.0258 (3)	
H11	1.1170	0.4415	0.7155	0.031*	
C12	1.11166 (16)	0.57152 (10)	0.68749 (13)	0.0246 (3)	
C13	1.24183 (16)	0.58518 (10)	0.72991 (13)	0.0252 (3)	
C14	1.29543 (16)	0.66804 (10)	0.73119 (14)	0.0275 (3)	
H14	1.3836	0.6774	0.7589	0.033*	
C15	1.21964 (17)	0.73610 (10)	0.69203 (15)	0.0294 (4)	
H15	1.2566	0.7922	0.6926	0.035*	
C16	1.08972 (17)	0.72399 (10)	0.65159 (14)	0.0288 (3)	
C17	1.03685 (16)	0.64216 (10)	0.64830 (14)	0.0262 (3)	
H17	0.9490	0.6336	0.6192	0.031*	
C18	1.44150 (17)	0.52479 (11)	0.81125 (16)	0.0328 (4)	
H18A	1.4763	0.4690	0.8361	0.049*	
H18B	1.4415	0.5643	0.8770	0.049*	
H18C	1.4978	0.5481	0.7504	0.049*	
C19	0.89413 (19)	0.78713 (12)	0.56997 (19)	0.0415 (4)	
H19A	0.8576	0.8436	0.5503	0.062*	
H19B	0.8368	0.7592	0.6264	0.062*	
H19C	0.8983	0.7518	0.5002	0.062*	

### Atomic displacement parameters (Å^2^)

**Table:**

	*U*^11^	*U*^22^	*U*^33^	*U*^12^	*U*^13^	*U*^23^
S1	0.0323 (2)	0.0271 (2)	0.0610 (3)	−0.00414 (17)	−0.0128 (2)	−0.0060 (2)
O1	0.0306 (6)	0.0158 (5)	0.0640 (8)	0.0014 (5)	−0.0106 (6)	0.0012 (5)
O2	0.0364 (7)	0.0202 (5)	0.0534 (8)	0.0041 (5)	−0.0167 (6)	0.0012 (5)
O3	0.0260 (6)	0.0242 (6)	0.0465 (7)	−0.0029 (5)	−0.0060 (5)	−0.0024 (5)
O4	0.0380 (7)	0.0223 (6)	0.0629 (9)	−0.0045 (5)	−0.0123 (6)	0.0066 (6)
N1	0.0214 (6)	0.0185 (6)	0.0287 (6)	−0.0005 (5)	−0.0028 (5)	0.0002 (5)
N2	0.0250 (7)	0.0158 (6)	0.0307 (7)	−0.0010 (5)	−0.0004 (5)	0.0005 (5)
C1	0.0230 (8)	0.0185 (7)	0.0312 (8)	−0.0009 (6)	−0.0002 (6)	0.0016 (6)
C2	0.0249 (8)	0.0167 (7)	0.0270 (7)	0.0005 (6)	−0.0025 (6)	−0.0014 (5)
C3	0.0274 (8)	0.0186 (7)	0.0288 (8)	0.0006 (6)	−0.0017 (6)	−0.0006 (6)
C4	0.0244 (8)	0.0200 (7)	0.0300 (8)	−0.0015 (6)	0.0008 (6)	−0.0011 (6)
C5	0.0242 (8)	0.0241 (7)	0.0309 (8)	0.0020 (6)	−0.0053 (6)	0.0034 (6)
C6	0.0285 (9)	0.0318 (9)	0.0419 (10)	0.0039 (7)	−0.0002 (7)	−0.0008 (7)
C7	0.0322 (9)	0.0160 (7)	0.0427 (9)	−0.0018 (6)	0.0037 (7)	0.0029 (6)
C8	0.0402 (10)	0.0252 (8)	0.0594 (12)	0.0003 (7)	0.0068 (9)	−0.0097 (8)
C9	0.0255 (8)	0.0202 (7)	0.0293 (8)	0.0011 (6)	−0.0020 (6)	−0.0024 (6)
C10	0.0262 (8)	0.0202 (7)	0.0299 (8)	−0.0005 (6)	−0.0010 (6)	−0.0021 (6)
C11	0.0270 (8)	0.0207 (7)	0.0298 (8)	−0.0001 (6)	0.0009 (6)	−0.0033 (6)
C12	0.0260 (8)	0.0220 (7)	0.0260 (7)	−0.0039 (6)	0.0029 (6)	−0.0045 (6)
C13	0.0254 (8)	0.0228 (7)	0.0275 (8)	−0.0009 (6)	0.0030 (6)	−0.0036 (6)
C14	0.0254 (8)	0.0271 (8)	0.0300 (8)	−0.0066 (6)	0.0018 (6)	−0.0054 (6)
C15	0.0313 (9)	0.0226 (7)	0.0345 (8)	−0.0091 (6)	0.0034 (7)	−0.0039 (6)
C16	0.0328 (9)	0.0224 (7)	0.0314 (8)	−0.0021 (6)	0.0013 (7)	0.0000 (6)
C17	0.0251 (8)	0.0230 (7)	0.0305 (8)	−0.0038 (6)	−0.0003 (6)	−0.0008 (6)
C18	0.0264 (8)	0.0320 (9)	0.0398 (9)	−0.0031 (7)	−0.0051 (7)	0.0009 (7)
C19	0.0365 (10)	0.0292 (9)	0.0587 (12)	−0.0017 (7)	−0.0097 (9)	0.0094 (8)

### Geometric parameters (Å, °)

**Table:**

S1—C4	1.6516 (16)	C8—H8A	0.9800
O1—C1	1.2160 (19)	C8—H8B	0.9800
O2—C3	1.2207 (19)	C8—H8C	0.9800
O3—C13	1.3582 (19)	C9—C10	1.427 (2)
O3—C18	1.430 (2)	C9—H9	0.9500
O4—C16	1.368 (2)	C10—C11	1.351 (2)
O4—C19	1.422 (2)	C10—H10	0.9500
N1—C4	1.3815 (19)	C11—C12	1.453 (2)
N1—C1	1.4065 (19)	C11—H11	0.9500
N1—C5	1.4791 (19)	C12—C17	1.405 (2)
N2—C4	1.385 (2)	C12—C13	1.407 (2)
N2—C3	1.396 (2)	C13—C14	1.400 (2)
N2—C7	1.4852 (19)	C14—C15	1.379 (2)
C1—C2	1.461 (2)	C14—H14	0.9500
C2—C9	1.360 (2)	C15—C16	1.394 (2)
C2—C3	1.465 (2)	C15—H15	0.9500
C5—C6	1.514 (2)	C16—C17	1.383 (2)
C5—H5A	0.9900	C17—H17	0.9500
C5—H5B	0.9900	C18—H18A	0.9800
C6—H6A	0.9800	C18—H18B	0.9800
C6—H6B	0.9800	C18—H18C	0.9800
C6—H6C	0.9800	C19—H19A	0.9800
C7—C8	1.512 (2)	C19—H19B	0.9800
C7—H7A	0.9900	C19—H19C	0.9800
C7—H7B	0.9900		
			
C13—O3—C18	118.65 (12)	H8A—C8—H8C	109.5
C16—O4—C19	117.25 (14)	H8B—C8—H8C	109.5
C4—N1—C1	124.56 (13)	C2—C9—C10	130.09 (15)
C4—N1—C5	119.26 (13)	C2—C9—H9	115.0
C1—N1—C5	116.18 (12)	C10—C9—H9	115.0
C4—N2—C3	124.38 (13)	C11—C10—C9	119.24 (15)
C4—N2—C7	119.65 (13)	C11—C10—H10	120.4
C3—N2—C7	115.81 (13)	C9—C10—H10	120.4
O1—C1—N1	119.92 (14)	C10—C11—C12	128.08 (15)
O1—C1—C2	123.75 (14)	C10—C11—H11	116.0
N1—C1—C2	116.32 (13)	C12—C11—H11	116.0
C9—C2—C1	123.71 (14)	C17—C12—C13	119.00 (14)
C9—C2—C3	117.04 (14)	C17—C12—C11	122.30 (15)
C1—C2—C3	119.24 (13)	C13—C12—C11	118.70 (14)
O2—C3—N2	119.86 (14)	O3—C13—C14	123.75 (15)
O2—C3—C2	123.26 (14)	O3—C13—C12	116.31 (13)
N2—C3—C2	116.89 (13)	C14—C13—C12	119.94 (15)
N1—C4—N2	117.14 (13)	C15—C14—C13	119.73 (15)
N1—C4—S1	121.84 (12)	C15—C14—H14	120.1
N2—C4—S1	121.01 (11)	C13—C14—H14	120.1
N1—C5—C6	111.71 (13)	C14—C15—C16	121.12 (14)
N1—C5—H5A	109.3	C14—C15—H15	119.4
C6—C5—H5A	109.3	C16—C15—H15	119.4
N1—C5—H5B	109.3	O4—C16—C17	125.20 (16)
C6—C5—H5B	109.3	O4—C16—C15	115.32 (14)
H5A—C5—H5B	107.9	C17—C16—C15	119.48 (15)
C5—C6—H6A	109.5	C16—C17—C12	120.71 (15)
C5—C6—H6B	109.5	C16—C17—H17	119.6
H6A—C6—H6B	109.5	C12—C17—H17	119.6
C5—C6—H6C	109.5	O3—C18—H18A	109.5
H6A—C6—H6C	109.5	O3—C18—H18B	109.5
H6B—C6—H6C	109.5	H18A—C18—H18B	109.5
N2—C7—C8	111.72 (14)	O3—C18—H18C	109.5
N2—C7—H7A	109.3	H18A—C18—H18C	109.5
C8—C7—H7A	109.3	H18B—C18—H18C	109.5
N2—C7—H7B	109.3	O4—C19—H19A	109.5
C8—C7—H7B	109.3	O4—C19—H19B	109.5
H7A—C7—H7B	107.9	H19A—C19—H19B	109.5
C7—C8—H8A	109.5	O4—C19—H19C	109.5
C7—C8—H8B	109.5	H19A—C19—H19C	109.5
H8A—C8—H8B	109.5	H19B—C19—H19C	109.5
C7—C8—H8C	109.5		
			
C4—N1—C1—O1	−172.42 (15)	C4—N2—C7—C8	88.82 (18)
C5—N1—C1—O1	6.6 (2)	C3—N2—C7—C8	−86.77 (18)
C4—N1—C1—C2	8.2 (2)	C1—C2—C9—C10	−2.7 (3)
C5—N1—C1—C2	−172.73 (13)	C3—C2—C9—C10	176.85 (15)
O1—C1—C2—C9	−11.3 (3)	C2—C9—C10—C11	−179.44 (16)
N1—C1—C2—C9	167.98 (14)	C9—C10—C11—C12	−179.07 (15)
O1—C1—C2—C3	169.12 (15)	C10—C11—C12—C17	0.0 (3)
N1—C1—C2—C3	−11.6 (2)	C10—C11—C12—C13	179.19 (15)
C4—N2—C3—O2	−173.69 (15)	C18—O3—C13—C14	−0.2 (2)
C7—N2—C3—O2	1.7 (2)	C18—O3—C13—C12	179.70 (14)
C4—N2—C3—C2	6.8 (2)	C17—C12—C13—O3	179.26 (13)
C7—N2—C3—C2	−177.84 (13)	C11—C12—C13—O3	0.1 (2)
C9—C2—C3—O2	5.6 (2)	C17—C12—C13—C14	−0.8 (2)
C1—C2—C3—O2	−174.83 (15)	C11—C12—C13—C14	−179.99 (14)
C9—C2—C3—N2	−174.93 (14)	O3—C13—C14—C15	−179.33 (14)
C1—C2—C3—N2	4.7 (2)	C12—C13—C14—C15	0.7 (2)
C1—N1—C4—N2	2.4 (2)	C13—C14—C15—C16	0.4 (2)
C5—N1—C4—N2	−176.56 (13)	C19—O4—C16—C17	−3.6 (3)
C1—N1—C4—S1	−177.59 (12)	C19—O4—C16—C15	176.54 (16)
C5—N1—C4—S1	3.42 (19)	C14—C15—C16—O4	178.46 (15)
C3—N2—C4—N1	−10.5 (2)	C14—C15—C16—C17	−1.4 (2)
C7—N2—C4—N1	174.31 (13)	O4—C16—C17—C12	−178.52 (15)
C3—N2—C4—S1	169.52 (12)	C15—C16—C17—C12	1.3 (2)
C7—N2—C4—S1	−5.67 (19)	C13—C12—C17—C16	−0.2 (2)
C4—N1—C5—C6	82.80 (17)	C11—C12—C17—C16	178.91 (15)
C1—N1—C5—C6	−96.28 (16)		
